# The Biodevelopment of Sexual Orientation: Beyond the Known Horizon

**DOI:** 10.1007/s10508-022-02506-1

**Published:** 2022-12-27

**Authors:** Wojciech Ł. Dragan, Monika Folkierska-Żukowska

**Affiliations:** 1 Institute of Psychology, Jagiellonian University, Ingardena Str 6, 30-060 Kraków, Poland; 2 Faculty of Psychology, University of Warsaw, Warsaw, Poland

This Commentary addresses the Target Article by VanderLaan et al. (2022) on the biodevelopment of same-sex sexual orientation. First, we would like to acknowledge that their work comprehensively reviews the most up-to-date knowledge about human sexual orientation. Crucially, it is an important review in that it highlights the growing evidence that non-heterosexual people, even within the most commonly-used sexual orientation categories, are not homogeneous but may be characterized by distinct biodevelopmental sub-groupings. In this Commentary, we would like to further elaborate on some of the issues raised by this review related to the conceptualization and biological underpinnings of sexual orientation. We also would like to expand the range of future research directions into the biodevelopment of same-sex sexual orientation based on recent advances in other research areas.

We agree with VanderLaan et al. (2022) that our understanding of sexual orientation biodevelopment is primarily a consequence of this construct's current conceptualization and measurement. Although there have been attempts to measure sexual orientation by measuring sexual arousal or other reactions (such as pupil dilation) in response to different sexual stimuli (Chivers et al., 2007; Ciardha et al., 2018; Rieger & Savin-Williams, 2012), no reliable objective measure of sexual orientation that is easy to use in a laboratory setting has yet been developed, and researchers usually depend on self-report questionnaires.^1^ While there have been many attempts to do justice to the multidimensional nature of sexual orientation, none can be considered perfect (see, e.g., Sell, 2007), and choosing an appropriate way to measure it remains a challenge. According to the Kinsey Institute (2022), there are over 200 different scales for measuring sexual orientation. Typically, researchers need to consider how to balance the level of nuance captured by a measure with the measure’s simplicity for respondents to fill out, while at the same time providing data that are easy to analyze.

When using self-report scales, it is currently considered best practice to measure the components of sexual orientation: behavior (sexual partners), sexual attractions (desires), and identity (Sell, 2007). Nevertheless, some researchers (especially in epidemiological studies) use behavioral definitions, sometimes even refraining from referring to sexual orientation per se and studying, for example, “men who have sex with men” (Center for Disease Control & Prevention, 2022). Despite efforts to create nuanced definitions of sexual orientation, the orientations most commonly referred to, both in everyday life and in research, are still heterosexuality, homosexuality, and bisexuality (with the last often omitted). It is still the case that few studies consider asexuality (Bogaert, 2006)—even though using only three categories of sexual orientation (especially when talking about a person’s identity) is probably insufficient.

Sexual fluidity and bisexuality/pansexuality both give rise to nonexclusive sexual attractions, but through different pathways—the first is a context-dependent capacity for change in attractions, and the second is a pattern of mixed attractions. Thus, when individuals report experiences of nonexclusive attractions, it can potentially stem from both bisexuality or sexual fluidity (Diamond, 2016; Diamond et al., 2020).

There is evidence that identities such as “mostly heterosexual” and “mostly homosexual” also constitute distinct sexual orientations (Savin-Williams & Vrangalova, 2013). Moreover, many individuals identify themselves with even more nuanced labels (e.g., pansexual, queer, fluid, demisexual; Galupo et al., 2015), which is of increasing interest to social scientists (e.g., Galupo et al., 2015; Oakley, 2016). It would be interesting to investigate the associations between these and biodevelopmental variables. However, research into biological determinants or correlates of sexual orientation has not yet caught up with these more nuanced categories, and it usually limits itself to the most basic categories. This is done, at least in part, for pragmatic reasons—investigating “extreme” sexual orientations is a good starting point, as it may facilitate the drawing of more clear-cut conclusions by limiting the number of variables. Nonetheless, investigating more nuanced sub-categories will hopefully follow as the next step.

From an essentialist perspective, it is hard not to ask whether these identity-related subdivisions and sexual fluidity are based in biology or social phenomena (or, most likely, some complex interplay between them). Some studies have broached this question by investigating different conceptualizations of sexual orientation in non-Western cultures (Gómez et al., 2018; Semenyna et al., 2017; Thurston et al., 2021; Vasey & VanderLaan, 2007). We believe studying the correlates of the newly-emerging labels in Western culture is also worthwhile.

Moreover, it may be worth studying not just sexual orientation alone but also putting more effort into understanding romantic orientation (the two can diverge in one individual, and the latter can also apply to asexual individuals; (Bogaert, 2015; Lund et al., 2016) as well as levels of sexual interest (with no sexual interest constituting asexuality; Bogaert, 2015). It is striking that most studies considering sexual orientation focus on sexual attractions and activity, ignoring people’s romantic and pair-bonding inclinations. Indeed, we could say that the word “sexual” in “sexual orientation” is where the answer hides, but we also cannot forget that the sexual minority activists’ slogan reads “love is love” rather than “sex is sex.”

However, we should not only consider adding additional parameters when describing sexual orientation variability but also try to move beyond the traditional, linear perspective. One possible direction for future studies might be the use of circular or circumplex models (e.g., Rogoza et al., 2021), which were recently utilized in research into personality and individual differences. This way of conceptualizing relationships between different variables has also been used in studies on values (Schwartz et al., 2013) and identity formation (Cieciuch & Topolewska, 2017). One of the advantages of circumplex models is the possibility of testing specific hypotheses regarding relationships with external variables. We think this may help disentangle the complexity of the relationship between sexual orientation and gender identity. We also believe that utilizing circumplex models in research on the biological bases of sexual orientation may shed light on the underpinnings of bisexuality and asexuality, which are rarely studied in this context. Interestingly, moving from linear to other forms of conceptualization and measurement of sexual orientation was suggested by Ganna et al. (2019) in the most extensive genome-wide association study (GWAS) on the topic to date. Their findings showed that, on the genetic level, there was no evidence for a single dimension of opposite-sex to same-sex preference, which is assumed by the most ubiquitous measures of sexual orientation, such as the Kinsey scale.

Regarding gender differences in the biological developmental underpinnings of sexual orientation, many studies suggest that this process differs between men and women. In one way, this seems logical—if we conceptualize sexual orientation as gynephilia and androphilia rather than same- or other-sex attraction, then it is intuitive that the two arise through different pathways, or at least taking different directions on the same path. However, this also suggests that the same mechanism would be responsible for gynephilia or androphilia, irrespective of sex. Thus, we may expect a kind of spectrum where, through the action of some mechanism (hormonal, genetic, or immunological), we end up with gynephilia on one extreme and androphilia on the other. However, this is also not straightforwardly supported by empirical evidence. For instance, if we take prenatal testosterone, the basic hypothesis is that low levels should result in androphilia and high levels in gynephilia. Meta-analyses seem to agree that there is a link between increased testosterone in women and gynephilia, but there does not appear to be a relationship between low prenatal testosterone and androphilia in men (Breedlove, 2017; Grimbos et al., 2010). Notably, these findings also beg the question of whether this link appears to be more evident for women simply because fewer studies on women have been published. It is often the case that more attempts at replicating a given result yield more inconsistent data. Indeed, as mentioned by VanderLaan et al. (2022), further attempts to replicate these studies seem to support the role of testosterone for male but not female sexual orientation. Second, as pointed out by VanderLaan et al., it is also worth noting here that there is evidence to suggest that both higher and lower levels of androgen activity during development could be related to male androphilia. This again seems to conflict with the expectation that sex hormones play a linear “two ends of the spectrum” role in sexual orientation development and suggests that, at least in the case of men, this relationship could be curvilinear (Bogaert & Hershberger, 1999; Skorska & Bogaert, 2017; Swift-Gallant et al., 2019b, 2021). It should be stressed that studies on the role of testosterone in the development of human sexual orientation largely rely on digit-ratio measurements, and the validity of these measures remains a matter of debate. Evidence from handedness studies also proposes such a curvilinear relationship: both left-handedness and extreme right-handedness have been reported to be associated with male androphilia (e.g.,Bogaert, 2007; Ellis et al., 2016; Kishida & Rahman, 2015; Lalumière et al., 2000). Some evidence suggests that, in men, gender expression and top/bottom sexual role may differentiate in response to exposure to high or low prenatal testosterone levels (e.g., Swift-Gallant et al., 2021). To date, we are unaware of any studies suggesting such a curvilinear relationship in women. Some evidence also suggests (although not conclusively) that women are more fluid regarding their sexual orientation than men (Diamond, 2016; cf. Katz-Wise & Hyde, 2015). Another clear example of such an “asymmetrical” influence on sexual orientation (that only concerns men and not women) is the fraternal birth order/immunological effect, which seems responsible for the development of androphilia in 15–29% of gay men (Blanchard & Bogaert, 2004). Last but not least, an extensive GWAS on sexual orientation found that loci associated with sexual orientation overlap only partially in men and women (Ganna et al., 2019).

With that being said, making strong claims about sex differences in the development of sexual orientation is difficult, as women are notoriously understudied in the context of sexual orientation. For instance, as of 2021, there were 26 published studies regarding the relationship of brain structure and function with sexual orientation (both postmortem and using neuroimaging techniques), of which only 7 included lesbians (Abé et al., 2021; Burke et al., 2017; Kranz & Ishai, 2006; Ponseti et al., 2006; Savic & Lindström, 2008; Sylva et al., 2013; Zeki & Romaya, 2010). Again, there is some new evidence to suggest that the biology of sexual orientation could be easier to pinpoint in women if only they were studied more often. A recent neuroimaging study used a machine learning approach to classify brains as male and female based on structural volumetric and diffusion tensor imaging data from an enormous sample of 18,645 individuals from the UK Biobank. It revealed that the cross-sex shift in brain structure was present in homosexual individuals of both sexes and was more significant in homosexual women than in homosexual men (Abé et al., 2021).

Interestingly, studying women in the context of male sexual orientation may also prove worthwhile. One of the hypotheses about the origin of male sexual orientation supported by the strongest indirect and direct evidence is the aforementioned fraternal birth order effect, which results from an in utero maternal immune response to male-specific antigens that are produced by male fetuses. As previously mentioned, this mechanism appears to explain sexual orientation in a subset of gay men (Blanchard & Bogaert, 2004; Cantor et al., 2002). There have not been many studies directly investigating this mechanism, but those conducted provided clear evidence to support it. Piper et al. (2007) reported that specific cytotoxic lymphocytes sensitive to a protein coded on the Y-chromosome were found in about 37% of women who had had one male pregnancy and as many as 50% of women who had had at least two male pregnancies. Bogaert et al. (2018) identified proteins coded on the Y-chromosome that are expressed in the brain and could be attacked by maternal antibodies. They compared immunization toward these proteins in mothers of gay and heterosexual men, considering mothers’ total number of male pregnancies. One of the antigens (a sign of immunization) was present in significantly larger quantities in mothers of gay men, with mothers of gay men who had mostly older brothers being the most immunized.

It is interesting to consider these results in the context of genetic research on male sexual orientation. Apart from the fact that sexual orientation is a complex trait, one other possible reason why genetic studies on male sexual orientation are unclear could be that there exist several biodevelopmental pathways leading to the same sexual orientation. However, in the case of some men, it may also be that it is not the gay men’s DNA but their mother's DNA that determines their sexual orientation. This may further “dilute” the results of genetic studies. Considering the possibility of indirect genetic effects seems only logical. Such studies would be challenging to perform but seem increasingly plausible in the world of huge DNA databases such as 23 and Me (e.g., Ganna et al., 2019). There is already some research into the role of mothers’ genetic makeup in determining their sons’ sexual orientation; for example, some evidence has been found for extreme skewing of *X* chromosome inactivation in mothers of homosexual men (Bocklandt et al., 2006), as well as research showing that male homosexuality may be associated with genetic selection for fecundity in their female relatives (Iemmola & Camperio Ciani, 2008). It seems worthwhile to continue this line of research.

The most extensive GWAS study on sexual orientation by Ganna et al. (2019) revealed that SNP-based heritability does not fully capture the heritability estimated using family-based methods. The problem of so-called missing heritability (Young, 2019) is typical for the genetics of complex traits. Many explanations for this issue have been proposed (e.g., Génin, 2020), including insufficient power of existing GWASs to detect rare variants or methodological issues related to estimating heritability. Height has always been given as an illustration of the detection of genes for complex traits. The early GWASs for height detected SNPs responsible for up to 5% of the phenotypic variance (as compared to the heritability of 80% estimated using twin studies; Gudbjartsson et al., 2008). A recent study by Yengo et al. (2022), using data from more than 5 million participants, identified SNPs that accounted for 40% of phenotypic variance in populations of European ancestry. Moreover, the same team of researchers (Wainschtein et al., 2022) found that the heritability for height estimated using the whole-genome sequencing data is 68%. The results of these studies suggest future directions for genetic studies on sexual orientation. Larger GWAS samples or using whole-genome sequencing may lead to the identification of rare variants related to sexual orientation and a more in-depth understanding of its biodevelopment.

Another area of genetic studies that may expand our knowledge on the topic is the use of polygenic scores (polygenic indexes, genome-wide scores). The polygenic score (PGS) reflects an individual’s genetic predisposition to a particular trait. It can be derived from the results of a GWAS study based on the weights of phenotype-associated alleles (Torkamani et al., 2018). There are several applications of PGS, with the main one being the use of the polygenic score as a predictor of a given phenotype or its correlates. For example, Abé et al. (2021) investigated relationships between polygenic risk score for same-sex behavior and cortical volumes in specific brain areas. However, polygenic scores can also be used in studying gene-environment interactions (GxE).

The investigation of sexual orientation with twin studies has generated consistent findings on gender differences in the parts of the phenotypic variance that can be attributed to the shared and non-shared environments. In males, a non-genetic part of the variance is typically explained solely by the non-shared environment, whereas in females, it can be attributed to both types of environment (see, e.g., Bailey et al., 2000). A non-shared environment may cover the impact of biological (but other than genetic) factors operating prenatally. Examples of shared environmental factors relevant to sexual orientation studies include family child-rearing styles, religious beliefs, and culture. Future GxE studies on sexual orientation should focus on different aspects of the environment, considering the effect of gender differences on the impact of shared and non-shared environments. GxE studies could also shed some light on possible interactions between different biological mechanisms (i.e., genetic, hormonal, immunological) that contribute to differences in sexual orientation. In their article, VanderLaan et al. (2022) mentioned that existing studies suggest the possibility that neurodevelopmental processes that shape sexual orientation can operate independently of one another. However, we disagree that this is plausible. It seems unlikely to us that a trait as multifactorial as sexual orientation could be the effect of only one biological factor. Interactions between different factors and processes are embedded in the developmental complexity of human behavior.

Moving forward, GxE studies on sexual orientation need more reliable proxy measures of the impact of prenatal androgens. As VanderLaan et al. (2022) point out, the most commonly-used marker of prenatal androgen exposure is the ratio of the length of the second digit to the fourth digit of one’s hand (2D:4D). The validity of the 2D:4D ratio, however, is contested (Hampson & Sankar, 2012). Lutchmaya et al. (2004) found that the ratio of testosterone to estradiol present in second-trimester amniotic fluid was negatively correlated with digit ratios for the right hand. The replication study by Richards et al. (2020a) did not confirm this finding. Similarly, recent study (Barrett et al., 2021) did not find a correlation between maternal sex hormone concentrations during pregnancy and the 2D:4D ratio of their children. Interestingly, the meta-analysis of the relationship between congenital adrenal hyperplasia and the 2D:4D ratio (Richards, et al., 2020b) revealed a significant association for the right hand in males and the left hand in females. Taken together, these findings suggest that the relationship between prenatal androgen exposure and 2D:4D may be more complex than it appears. More validation studies are needed—not only for the 2D:4D ratio but also for other potential biomarkers of the action of prenatal hormones on development.

Finally, we agree with VanderLaan et al. (2022) that studies on the biodevelopment of sexual orientation have to move beyond already known mechanisms and factors. In the seminal work by Swift-Gallant, et al. (2019a) that used the latent profile analysis approach, most non-heterosexual participants fit the group that showed no evidence of biomarkers. This strongly suggests that something is missing. The results of Swift-Gallant et al. (2019a) may stem from the interactions of biological mechanisms that have already been identified (e.g., genes, hormones, immune response) or from additional ones. Some possible additional mechanisms include epigenetic influences (Ngun & Vilain, 2014) or alternative immunological factors (Blanchard, 2012). Large genotypic–phenotypic databases and the use of machine learning and data mining methods can reveal other as yet unknown biological mechanisms and push our understanding of the biodevelopment of sexual orientation beyond the known horizon.

## Footnotes

1 One objective measure related to sexual orientation is penile/vaginal plethysmography (Diamond & Rosky, 2016). However, there are several difficulties associated with this measure: it seems only reliable in individuals with penises (Bailey, 2009), which is due to the nonspecific patterns of arousal found in women (Chivers et al., 2004); performing it presents difficulties of many kinds (laboratory resources, participants willing to actually partake in such assessment, etc.); and crucially, while it is definitely a reliable measure of sexual arousal, it is not clear that it is a reliable measure of sexual orientation, which is believed that sexual orientation is a multidimensional trait (Sell, 1996, 2007).
